# Effect of Low Intensity Transcranial Ultrasound Stimulation on Neuromodulation in Animals and Humans: An Updated Systematic Review

**DOI:** 10.3389/fnins.2021.620863

**Published:** 2021-04-14

**Authors:** Taewon Kim, Christine Park, Pratik Y. Chhatbar, Jody Feld, Brian Mac Grory, Chang S. Nam, Pu Wang, Mengyue Chen, Xiaoning Jiang, Wuwei Feng

**Affiliations:** ^1^Department of Neurology, Duke University School of Medicine, Durham, NC, United States; ^2^Physical Therapy Division, Department of Orthopaedic Surgery, Duke University School of Medicine, Durham, NC, United States; ^3^Fitts Department of Industrial and Systems Engineering, North Carolina State University, Raleigh, NC, United States; ^4^Department of Rehabilitation Medicine, Seventh Affiliated Hospital, Sun Yat-sen University, Shengzhen, China; ^5^Department of Mechanical and Aerospace Engineering, North Carolina State University, Raleigh, NC, United States

**Keywords:** ultrasound, low intensity transcranial ultrasound stimulation, non-invasive brain stimulation, neuromodulation, human, animal

## Abstract

**Background:** Although low-intensity transcranial ultrasound stimulation (LI-TUS) has received more recognition for its neuromodulation potential, there remains a crucial knowledge gap regarding the neuromodulatory effects of LI-TUS and its potential for translation as a therapeutic tool in humans.

**Objective:** In this review, we summarized the findings reported by recently published studies regarding the effect of LI-TUS on neuromodulation in both animals and humans. We also aim to identify challenges and opportunities for the translation process.

**Methods:** A literature search of PubMed, Medline, EMBASE, and Web of Science was performed from January 2019 to June 2020 with the following keywords and Boolean operators: [transcranial ultrasound OR transcranial focused ultrasound OR ultrasound stimulation] AND [neuromodulation]. The methodological quality of the animal studies was assessed by the SYRCLE's risk of bias tool, and the quality of human studies was evaluated by the PEDro score and the NIH quality assessment tool.

**Results:** After applying the inclusion and exclusion criteria, a total of 26 manuscripts (24 animal studies and two human studies) out of 508 reports were included in this systematic review. Although both inhibitory (10 studies) and excitatory (16 studies) effects of LI-TUS were observed in animal studies, only inhibitory effects have been reported in primates (five studies) and human subjects (two studies). The ultrasonic parameters used in animal and human studies are different. The SYRCLE quality score ranged from 25 to 43%, with a majority of the low scores related to performance and detection bias. The two human studies received high PEDro scores (9/10).

**Conclusion:** LI-TUS appears to be capable of targeting both superficial and deep cerebral structures to modulate cognitive or motor behavior in both animals and humans. Further human studies are needed to more precisely define the effective modulation parameters and thereby translate this brain modulatory tool into the clinic.

## Introduction

Transcranial ultrasound stimulation (TUS) is a novel non-invasive brain stimulation (NIBS) technique that is currently in a rapid phase of investigation as a neuromodulation tool. TUS uses acoustic energy that modulates central neural circuits and induces changes in neuronal activity in both the central and peripheral nervous systems (Blackmore et al., 2019). The fundamental parameter of TUS is intensity (W/cm^2^), defined as power transferred per unit area with the average intensity of an individual pulse (spatial-peak pulse-average, I_SPPA_) and over the total time-averaged intensity (spatial-peak temporal-average, I_SPTA_), which is commonly reported for time-periodic or steady state fields. Neuromodulation using high intensity TUS is associated with I_SPPA_ >200 W/cm^2^ and an accumulation of thermal energy. Whereas LI-TUS (I_SPPA_ <100 W/cm^2^) is not likely associated with an accumulation of significant thermal energy over the scalp or inside the brain (Tyler et al., 2018). The parameters optimization has been tried through modification of intensity, frequency, sonication duration (SD), duty cycle (DC), pulse duration (PD), and pulse repetition frequency (PRF).

LI-TUS has a high potential to be a viable neuromodulation tool to make changes in neural activity in targeted tissues in both the central and peripheral nervous systems (Darrow, 2019). However, the mechanisms underlying the neuromodulatory effect of LI-TUS are complexed and remain unclear despite numerous investigations in the animal models. The putative mechanisms have shown that ultrasound waves can effectively interact with biological tissues and transmit acoustic energy that then can induce mechanical and thermal bioeffects (Blackmore et al., 2019). A leading hypothesis posits that the acoustic radiation force may alter the conductance and channel activity of the mechanosensitive ion channels. Specifically, the mechanical force from the acoustic energy may induce cell membrane sonoporation that can alter membrane permeability and hence the electrochemical properties of cells. In addition to the mechanical effects, another possible mechanism of the neuromodulatory effects of LI-TUS is its thermal effects in tissues. A temperature rises caused by LI-TUS lead to evoke heat transfers which alter in the membrane properties as a result in depolarization (Tyler et al., 2018; Kamimura et al., 2020; Rabut et al., 2020).

Compared to other NIBS modalities, LI-TUS offers greater spatial resolution and focused penetration depth, which potentially opens access to subcortical brain structures (Di Biase et al., 2019). Evidence of LI-TUS induced neuromodulation of cortical and deep structures of the brain relies heavily on data obtained from animal studies (Wang et al., 2019b). In human studies, LI-TUS has been administered primarily in the primary somatosensory (S1) and primary motor (M1) cortices with limited success (Legon et al., 2014, 2018). Deep brain structures such as the somatosensory nuclei of the thalamus have been targeted with changes noted in the somatosensory evoked potential (Fomenko et al., 2018; Di Biase et al., 2019).

The field of LI-TUS is rapidly progressing with numerous publications publishing every quarter, it is critical to summarize emerging evidence on the status of this brain modulatory tool. In this review, we aim to provide an updated overview of initial version of systematic review regarding the LI-TUS for neuromodulation in both human and animal research since our initial review on this topic *that summarized the finding prior to 2019* (Wang et al., 2019b).

## Methods

### Literature Search Strategy

We followed the Preferred Reporting Items for Systematic Reviews and Meta-analyses (PRISMA) criteria (Moher et al., 2009). PubMed, Medline, EMBASE, and Web of Science databases were searched from January 2019 to June 2020 using the following keywords and Boolean operators: [transcranial ultrasound OR transcranial focused ultrasound OR ultrasound stimulation] AND [neuromodulation] (see Supplementary Table 1 for additional details on search terms). Language limitations were not imposed to ensure the inclusion of published manuscripts in other languages. In addition, two recently published systematic review articles were reviewed to exclude studies that already reviewed by Wang et al. (2019b) and Di Biase et al. (2019).

### Study Selection and Data Extraction

Studies that met the inclusion criteria modeled after the PICOS framework (Methley et al., 2014) were included for review: (1) Population (P): studies had to be conducted in humans or animals; (2) Intervention (I): studies had to use either focused or unfocused transcranial ultrasound stimulation to modulate neuronal activity or brain function; (3) Comparison (C): studies included with or without control conditions to investigate the effect of LI-TUS; (4) Outcomes (O): studies had to provide outcomes with at least one quantitative measure for assessing the effect of LI-TUS for human or animal neuromodulation; (5) Study type (S): studies were designed as experimental studies that included either uncontrolled or controlled trials. Studies were excluded if they met any of the following criteria: (1) data that was not published in a peer-reviewed journal; (2) review or commentary studies; (3) technical, computational, or diagnostic studies that do not consider the therapeutic effects of LI-TUS; (4) physiologic studies (i.e., studies probing mechanisms in non-living biological tissue). The following information was extracted from each article: first author's name, country, and publication year, specific population, stimulated brain region, experimental design, characteristics and parameters of ultrasound stimulation, method of outcome assessment, neuromodulatory effect, and major findings.

### Methodological Quality Assessments

The Systematic Review Center for Laboratory Animal Experimentation (SYRCLE)'s Risk of Bias tool (Hooijmans et al., 2014) and the Physiotherapy Evidence Database (PEDro) scale (Bhogal et al., 2005) were referenced for animal and human studies, respectively, to assess the methodological quality of the included studies. Uncontrolled or single-case trial studies were assessed by the National Institutes of Health (NIH) quality assessment tool (National Heart and Institute, 2019). Three rating qualities (Good, Fair and Poor) determined the degree of risk of bias (see details in Supplementary Materials). Two of the authors (TK, CP) independently conducted the risk of bias assessments for all included studies. Inconsistency was checked by the senior author (WF).

## Results

### Literature Search

**Figure 1:**
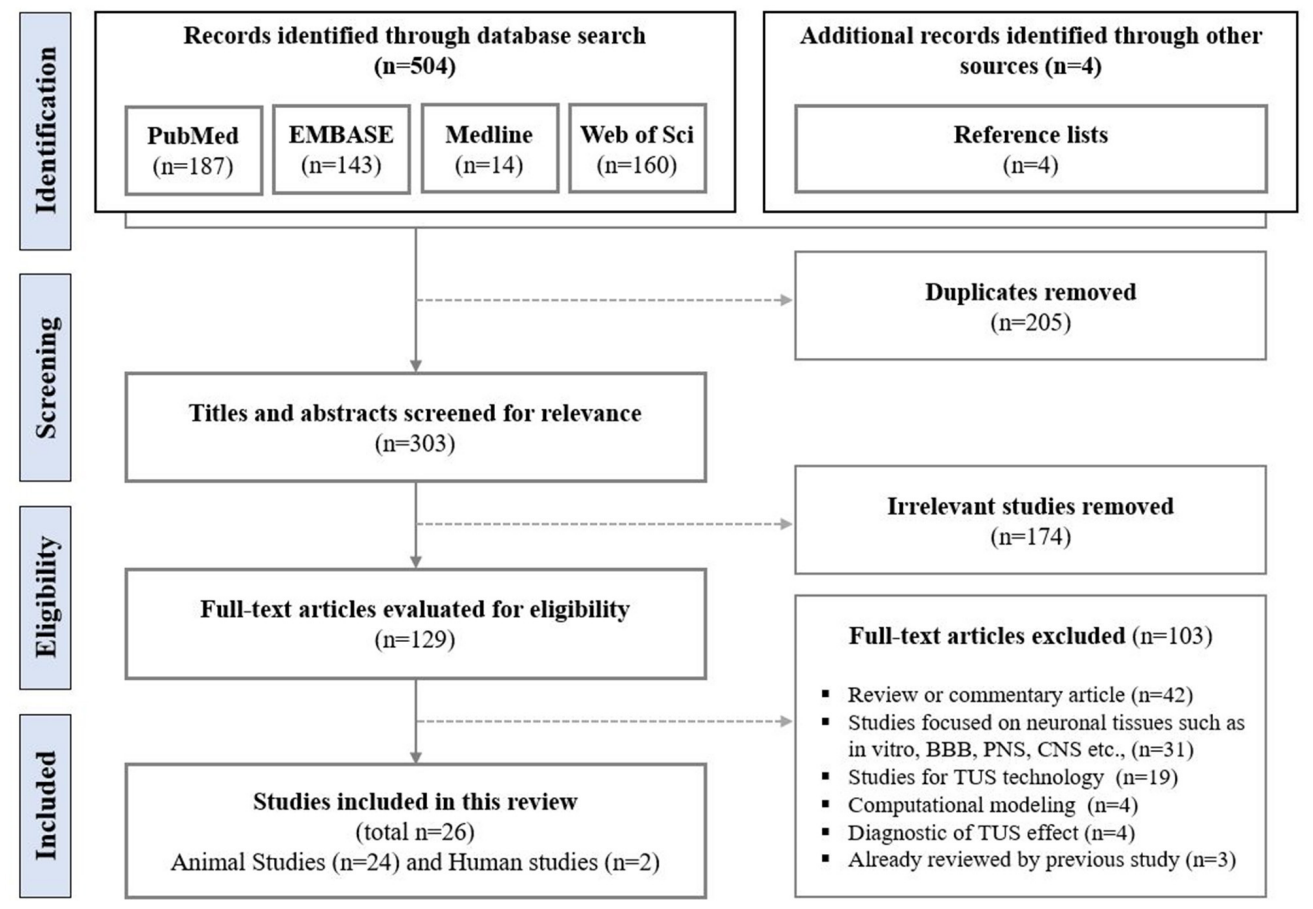
Flowchart of literature search.

The initial search from PubMed (*n* = 187), Medline (*n* = 14), EMBASE (*n* = 143), and Web of Science (*n* = 160) identified 504 studies. Additionally, four relevant studies were retrieved from a search of the reference lists of the final selected studies. After screening, 205 duplicate studies and 174 studies with irrelevant and/or insufficient data were removed. Out of the remaining 129 publications, 103 studies were excluded during the full-text assessment for the following reasons: commentary (*n* = 42); technical, computational, and/or diagnostic (*n* = 27), biological (*n* = 31), and previously reviewed in recently published systematic reviews (*n* = 3). A final total of 26 publications (24 animal studies and two human studies) were included in this review (Figure 1).

### Neuromodulatory Effect of TUS in Animals

**Figure 2:**
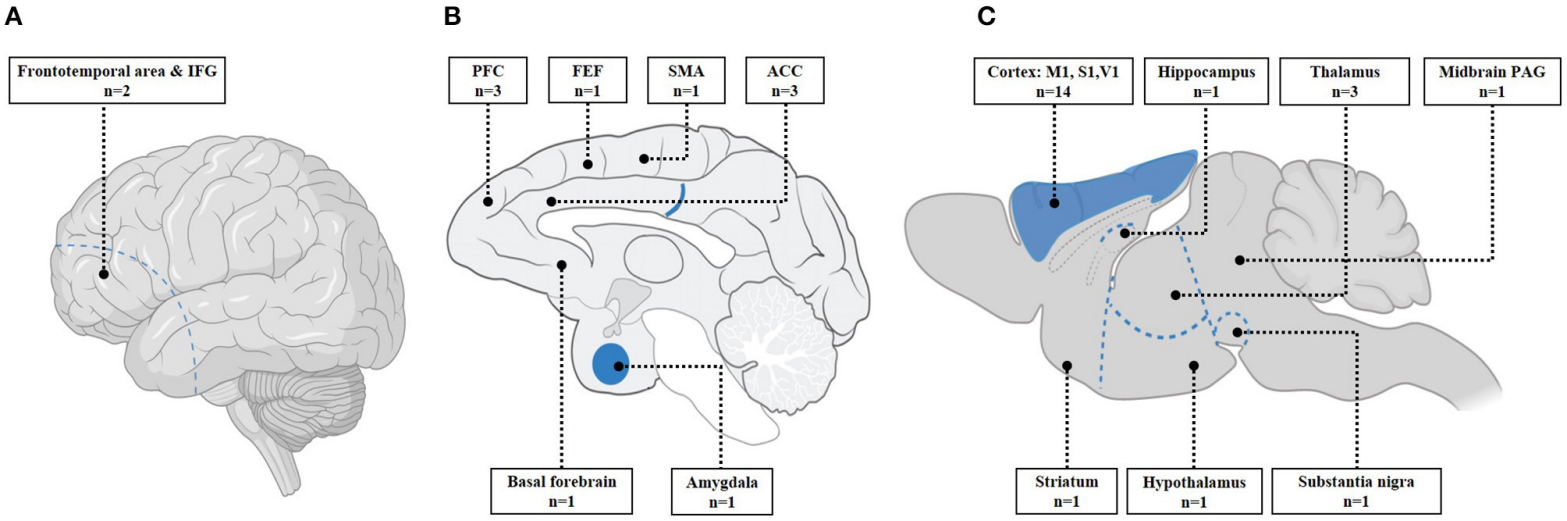
Stimulated regions; **(A)** left view of human brain; **(B)** midsagittal view of macaque brain; **(C)** midsagittal view of mice brain. This image displays targeted regions of mice studies and also includes one sheep study that stimulated at M1 and thalamus. IFG, inferior frontal gyrus; PFC, prefrontal cortex; FEF, frontal eye fields; SMA, supplementary motor area; ACC, anterior cingulate cortex; M1, primary motor cortex; S1, Somatosensory cortex; V1, visual cortex; PAG, periaqueductal gray. **(A,C)** was adapted from “human and mice brain icons,” by BioRender.com (2020). Retrieved from: https://app.biorender.com/biorender-templates. **(B)** was adapted from Raper et al. (2014).

Of the 24 animal studies, there were 18 with healthy animals, four with Parkinson's disease animal models, and two with epilepsy animal models. Three different animals (rodents, sheep, and macaque) were used including six non-human primate studies. LI-TUS was applied on various regions of animal brain (Figure 2) with different combinations of parameters (Table 1). Seven studies looked at stimulation of the M1 in healthy mice and found that LI-TUS induced changes in the activity of M1 that correlated to the success rate of the motor response (Kim et al., 2019b; Wang et al., 2019d) in electromyography (EMG), local field potential (LFP) (Wang et al., 2019c,d; Yuan et al., 2020), and cortical blood flow (Yuan et al., 2020). Specifically, EMG signals from the triceps muscles of forelimbs (Cui et al., 2019, 2020), the right distal forelimb (Wang et al., 2019a), bilateral whiskers, and tail (Wang et al., 2019d; Yuan et al., 2020) were examined for motor response changes in M1 after LI-TUS. EMG response latency was significantly decreased with higher acoustic pressure of 0.12 and 0.25 MPa (Cui et al., 2019, 2020) while EMG amplitudes were increased with higher LI-TUS intensity (Wang et al., 2019a; Yuan et al., 2020). LI-TUS with intensity at 1.1 W/cm^2^ (*I*_SPPA_) over M1 was able to elicit the greatest motor response in bilateral whiskers and tail and resulted in an increase in the peak values of cortical blood flow and neural activity responses (Yuan et al., 2020). The entropy of LFPs in M1 was induced at the time of LI-TUS with a range of parameters from 0.2 to 1.1 W/cm^2^ (*I*_SPPA_); SD: 100–400 ms, DC: from 10 to 40%) at 0–0.5 s (Wang et al., 2019c).

**Table 1:** Characteristics of studies included.

**Study**	**Subjects**	**Stimulated brain region**	**Study design**	**Protocol of TUS**	**Outcome measure**	**Modulatory effect**	**Major findings**
**CORTICAL AREA: FRONTAL LOBE**							
Cui et al. (2019) China	Normal mice (*n* = 20)	Primary motor cortex (M1)	Between-subjects with TUS and MBs-TUS	Frequency: 0.62 MHz Intensity: 0.48 and 2.11 W/cm^2^(*I*_SPPA_) PRF:250 Hz DC: 50% PD:2 ms SD: 400 ms	EMG (from the triceps muscles of forelimbs) Immunofluorescence	Excitatory	(1)TUS and TUS with MB at the motor cortex evoked a motor response and the EMG response latency was significantly decreased by higher acoustic pressure used with 0.12 MPa and 0.25 MPa. (2) TUS with MB showed greater neuronal activity and success rates of motor response than TUS.
Cui et al. (2020) China	Normal mice (*n* = 6)	Primary motor cortex (M1)	Between-subjects with TUS and MBs-TUS	Frequency: 0.62 MHz Intensity: 0.48 and 2.11 W/cm^2^(*I*_SPPA_) PRF:250 Hz DC: 50% PD: 2 ms SD: 400 ms	EMG (from the triceps muscles of forelimbs) Immunofluorescence	Excitatory	(1) Greater neural activity was induced by higher acoustic pressure used with 0.12 and 0.25 MPa in the TUS and TUS with MB. (2) TUS with MB showed greater neuronal activity and success rates of motor response than TUS.
Kim et al. (2019b) S. Korea	Normal, freely moving awake mice (*n* = 4)	Primary motor cortex (M1)	Within-subjects with sham control groups	Frequency: 0.183 MHz Intensity: 0.53 W/cm^2^(*I*_SPPA_) PRF: 200 Hz DC: 90% PD: 4.5 ms SD: 200 ms	EMG	Excitatory	TUS of the motor cortex evoked the success rate of motor responses measured by adjusting the AC voltage compared to control experiments.
Wang et al. (2019a) China	Normal mice (*n* = 16)	Primary motor cortex (M1)	Between-subjects with TMAS	Frequency: 0.5 MHz Intensity: ≤ 0.289 W/cm^2^(*I*_SPTA_) PRF: 1 kHz DC: 60% PD: 0.6 ms SD: 400 ms	EMG (from the right distal forelimb)	Excitatory	TUS induced EMG amplitudes that increased intensity showed greater EMG response.
Wang et al. (2019c) China	Normal mice (*n* = 24)	Primary motor cortex (M1)	Between-subjects with sham control	Frequency: 0.5 MHz Intensity: 0.2–1.1 W/cm^2^(*I*_SPPA_) PRF: 1 kHz DC: 10–40% PD: 0.1–0.4 ms SD: 100–400 ms	Neuronal activity: LFPs	Excitatory	TUS altered the relative power and entropy of neural oscillations in the motor cortex associated with different intensity and SD at 0–0.5 s stimulation duration.
Wang et al. (2019d) China	Normal mice (*n* = 21)	Primary motor cortex (M1)	Uncontrolled trial	Frequency: 0.5 MHz Intensity: 0.2–1.1 W/cm^2^(*I*_SPPA_) PRF: 1 kHz DC: 20–50% PD: 0.2–0.5 ms SD: 400 ms	LFPs and EMG using phase locking value analysis	Excitatory	TUS induced successful motor responses that were associated with varied parameters and correlated with particular frequency bands (5-150 Hz) of cortico-muscular synchronization. Alpha and beta synchronization appeared at 0.20 and 0.40 W/cm^2^(*I*_SPPA_), while gamma synchronization occurred at 0.80 and 1.10 W/cm^2^(*I*_SPPA_) stimulation.
Wang et al. (2020a) China	PD mice model (*n* = 77)	Primary motor cortex (M1)	Between-subjects with three control groups	Frequency: 0.5 MHz Intensity: 5.1 W/cm^2^(*I*_SPPA_) PRF: 1 kHz DC: 5% PD: 0.05 ms SD: 50 ms	LFPs	Excitatory	TUS directly induced the excitability of the M1 in PD mice.
Yuan et al. (2020) China	Normal mice (*n* = 29)	Primary motor cortex (M1)	Within-subjects uncontrolled trial	Frequency: 0.5 MHz Intensity: 0.2, 0.4, 0.8, and 1.1 W/cm^2^(*I*_SPPA_) PRF: 1 kHz DC: 10, 20, 30, 40% PD: 0.1, 0.2, 0.3, and 0.4 ms SD: 50, 100, 200, 300, 400 ms	EMG (from bilateral whisker and tail), LFP and CBF	Excitatory	TUS coupled with 1.10 W/cm^2^(*I*_SPPA_) intensity induced the highest motor behavior, neural activity and cortical hemodynamic responses reported by these measurements.
Zhou et al. (2019a) China	PD mice model (*n* = 32)	Primary motor cortex (M1)	Between-subjects with control, PD, sham + PD (*n* = 8 per group)	Frequency: 0.8 MHz Intensity: 760 mW/cm^2^(*I*_SPPA_) PRF: 100 Hz DC: 10% PD: 1 ms SD: 6-s	Behavioral: open field and pole test Immunofluorescence	Excitatory	(1) TUS in PD group showed superior behavioral performance compared to sham after 4 days of TUS at M1. (2) TUS induced increase of c-Fos positive cells in M1.
Verhagen et al. (2019) UK	Healthy macaques (*n* = 3)	Supplementary motor area (SMA) Frontal polar cortex (FPC)	Within-subjects with sham control groups	Frequency: 0.25 MHz Intensity: 7.2 W/cm^2^(*I*_SPTA_) for SMA Intensity: 9.5 W/cm^2^(*I*_SPTA_) for FPC PRF: 10 Hz DC: 30% PD: 30 ms SD: 40- s	Offline resting-state fMRI (T3)	Inhibitory	(1) TUS over SMA enhanced the coupling between SMA activity and activity in proximal areas, but reduced coupling between SMA and less closed regions. (2) TUS over FPC enhanced the connectivity between FPC and dorsomedial and lateral PFC and default mode network, but reduced connectivity with other regions in PFC and lateral orbitofrontal cortex.
Verhagen et al. (2019) UK	Healthy macaques (*n* = 3)	Frontal polar cortex (FPC)	Replication of TUS effects on FPC above	Intensity: 9.5 W/cm^2^(*I*_SPTA_) Other parameters were same as above	Offline resting-state fMRI (T3)	Inhibitory	TUS effects on FPC was the same as seen in experiment 2.
Kubanek et al. (2020) USA	Healthy macaques (*n* = 2)	Both frontal eye fields (FEF)	Within-subjects with active control region (Primary motor cortex)	Frequency: 0.27 MHz Intensity: 11.6 W/cm^2^(*I*_SPPA_) PRF: 500 Hz DC: 50% PD: 1 ms SD: 300 ms	Behavior: choice task	Indeterminable	(1) TUS influenced the animals' decisions in the contralateral direction. That is, targeting the left FEF increased the proportion of rightward choices, whereas, stimulation of the right FEF increased the proportion of leftward choices. (2) TUS at M1 did not elicit significant biases in choice behavior. This active control region provided regional specific effect of TUS.
Folloni et al. (2019) UK	Healthy macaques (TUS: *n* = 3, sham: *n* = 9)	Anterior cingulate cortex (ACC)	Between-subjects with placebo-controlled	Frequency: 0.25 MHz Intensity: 5.63 W/cm^2^(*I*_SPTA_) PRF: 10 Hz DC: 30% PD: 30 ms SD: 40-s	Offline resting-state fMRI (T3)	Inhibitory	(1) TUS modulated neural activity in ACC. (2) After the repetitive TUS, ACC's activity coupling patterns was altered. (3) Repetitive TUS protocol had no auditory confound from TUS over ACC.
Fouragnan et al. (2019) UK	Healthy macaques (*n* = 4)	Anterior cingulate cortex (ACC)	Within-subjects with sham control	Frequency: 0.25 MHz PRF: 10 Hz DC: 30% PD: 30 ms SD: 40-s	Offline resting-state fMRI (T3) Behavior: counterfactual choice (decision making)	Inhibitory	(1) TUS significantly changed ACC activity. (2) TUS at ACC altered strength of connectivity from ACC with 3 other regions. (3) After TUS at ACC, behavior was perturbed with monkeys less able to move and showed less accuracy.
Zou et al. (2020) China	Acute epilepsy model of monkeys (*n* = 2)	Prefrontal motor cortex (PFC)	Within-subjects with sham control groups	Frequency: 0.75, 0.8 MHz Intensity: ≤ 1.43W/cm^2^(*I*_SPTA_) Intensity: 119.78 W/cm^2^(*I*_SPPA_) PRF: 500 Hz DC: 36% PD: 0.72 ms SD: 100 ms	Behavioral seizure (video-EEG recoding)	Inhibitory	TUS group significantly reduced the number of epileptic seizures compared to that in the sham group in monkeys with acute epilepsy.
**CORTICAL AREA: PARIETAL AND OCCIPITAL LOBE**							
Kim et al. (2019a) S. Korea	Normal mice (*n* = 10)	Somatosensory cortex (S1)	Within-subjects with sham control groups	Frequency: 8 MHz Intensity: 1.077 and 0.468 W/cm^2^(*I*_SPTA_) PRF: 1.5 kHz DC: 9.375% PD: 62.5 μs SD: 200 ms	Near infrared spectroscopy (NIRS)	Excitatory	(1) TUS induced cerebral hemodynamic changes that showed increase of HbO and decrease of RHb. (2) Increasing the intensity induced a greater increases hemodynamic changes in the mice.
Choi et al. (2019) S. Korea	Normal mice (*n* = 5)	Somatosensory cortex (S1) and visual cortex (V1)	Within- subjects uncontrolled trial	Frequency: 10 MHz Intensity: 0.662 W/cm^2^(*I*_SPTA_) PRF: 1.5 kHz (with 300 bursts) and 300 bursts with random pulse repetition DC: 50% PD: 333 μs SD: 200 ms	Simultaneous wide-field optical imaging	Excitatory	(1) TUS with a needle transducer at S1 and V1 increased brain activity which showed higher neuronal calcium signal levels than those in peripheral regions. (2) Both periodic and random PRF stimulated in V1 and only periodic in S1 suppressed cortical activities in auditory cortex (A1).
**SUBCORTICAL AREAS**							
Darrow et al. (2019) USA	Normal rats (*n* = 15)	Thalamus	Uncontrolled trial	Frequency: 3.2 MHz Intensity: 20 W/cm^2^(*I*_SPTA_) PRF: 500 Hz DC: 5–70% SD: 30-s	Somato sensory evoked potentials (SSEP)	Inhibitory	(1) TUS suppressed the SSEP waveform when focused on the VPL contralateral to the stimulated tibial nerve. (2) Effect remained independent of duty cycle, peak pressure, or modulation frequency
Folloni et al. (2019) UK	Healthy macaques (TUS: *n* = 4) sham: *n* = 9	Amygdala	Between-subjects with sham control groups	Frequency: 0.25 MHz Intensity: 19.5 W/cm^2^(*I*_SPTA_) PRF: 10 Hz DC: 30% PD: 30 ms SD: 40-s	Offline resting-state fMRI (T3)	Inhibitory	(1) TUS modulated neural activity in amygdala. (2) After repetitive TUS, the amygdala's activity coupling patterns were disrupted. (3) Repetitive TUS protocol had no auditory confound from TUS over amygdala.
Pang et al. (2020) China	Aging mice (*n* = 44)	Hypothalamus	Between-subjects with sham control groups	Frequency: 1 MHz Intensity: 0.54 W/cm^2^(*I*_SPTA_) Intensity: 5.4 W/cm^2^(*I*_SPPA_) PRF: 1 kHz or 10 Hz DC: 10% PD: 0.1 ms or 10 ms SD: 1-s	Cognitive and motor behavior: Y maze test and grip strength test	Excitatory (indirectly measured)	TUS with 10 Hz of PRF group showed significantly superior movement and learning compared to TUS with 1,000 Hz of PRF and sham conditions.
Wang et al. (2020b) China	Normal mice (*n* = 27)	Midbrain periaqueductal gray (PAG)	Between-subjects with sham control	Frequency: 3.8 MHz Intensity: 140 mW/cm^2^(*I*_SPPA_) Intensity: 70 mW/cm^2^(*I*_SPTA_) PRF: 1 kHz DC: 50% PD: 0.5 ms SD: 400 ms	Defensive behavior: Passive avoidance, rat exposure, and open field test	Excitatory (indirectly measured)	TUS of PAG resulted in increased engagement of location-specific passive avoidance behavior and faster movement time compared to the sham stimulation group.
Xu et al. (2020) China	PD mice model (*n* = 48)	Striatum	Between-subjects with control	Frequency: 1 MHz Intensity: 0.1, 0.2, and 0.3 W/cm^2^ DC: continuous mode Ultrasound exposure time: 5, 10, 15 s	Behavioral: open field and pole test Dopamine (DA) content by HPLC coupled with electrochemical detection (six mice per group)	Excitatory	(1) Locomotor functions in PD mice were significantly improved after 10-day administration of non-focused TUS treatment at 0.30 W/cm^2^ over 5 min. (2) LI-TUS treatment with these parameters induced more dopamine (DA) release in the striatum compared to that in untreated PD mice.
Zhou et al. (2019b) China	Normal mice (*n* = 9) Parkinsonian mice (*n* = 30)	Substantia nigra	Between-subjects with TMAS and control group	Frequency: 1 MHz Intensity: 60 mW/cm^2^(*I*_SPTA_) PRF: 1 kHz DC: 20% PD: 200 μs SD: 120 ms	Behavioral: maze, open field, and MWM test; Electrophysiological: field excitatory postsynaptic potential (fEPSP)	Excitatory (improved synaptic plasticity)	(1) In health mice, TUS and TMAS condition showed greater behavioral performance than the control group, but not significantly different among the groups (*n* = 3 per group). (2) In PD mice, TUS and TAMS induced significant improvement in synaptic plasticity (long-term potentiation) which reflected in better spatial related forms of motor behavior performance than the control group.
**CORTICAL** **+** **SUBCORTICAL**							
Yoon et al. (2019) USA	Female sheep (*n* =10)	Left primary motor cortex (M1) and Left thalamic	Within-subjects with sham control groups	Frequency: 0.25 MHz Intensity: 15.8 and 18.2 W/cm^2^(*I*_SPPA_) PRF: 0.1–1 kHz DC: 20, 50, 70% and continuous mode PD: 0.5, 1, 2, and 3 ms SD: 200 ms	EMG (from both hind limbs) SEPs	Excitatory from EMG Inhibitory from SEP	(1) TUS at M1 and thalamic with 70% DC showed superior stimulation efficiency compared to other DC%. (2) 0.5 ms TBD in both regions resulted in the highest response rate compared to other TBD. (3) Greater motor response was observed in the contralateral hind leg to sonication of both the M1 and thalamus compared to ipsilateral side.
Chen et al. (2020) Taiwan	Epileptic rats (*n* = 76)	Cortex Hippocampus Thalamus	Between-subjects with sham control group	Frequency: 0.5 MHz Intensity:0.7–2.81W/cm^2^(*I*_SPTA_) PRF: 100 Hz DC: 8% or 30% Total exposure time:100 or 600 s	EEG Immunofluorescence	Inhibitory	(1) All varying TUS groups showed a significant decrease in number of epileptic EEG signal spikes compared to sham group at 10–15 min after PTZ injection. (2) TUS (DC 30%, 600 s) group showed significant increase in c-Fos-positive neurons in the cortex and hippocampus, but no differences were found in thalamus compared to sham group.
Khalighinejad et al. (2020) UK	Normal mice (*n* = 10)	ACC Basal forebrain (BF)	Within-subjects with active (parietal cortex) and sham control groups	Frequency: 0.25 MHz Intensity: (L) 6.4 and (R) 5.6 W/cm^2^(*I*_SPTA_) Intensity: (L) 21.2 and (R) 18.9 W/cm^2^(*I*_SPPA_) PRF: 10 Hz DC: 30% PD: 30 ms SD: 40- s	Behavior: decision about when to act; Offline resting-state fMRI (T3)	Inhibitory	(1) ACC was disrupted by TUS, which reflected in quicker act-time than both control conditions and TUS at BF. (2) After TUS at BF, activity coupling enhanced between the BF and ACC, but suppressed connectivity between BF and elsewhere.
**HUMAN STUDIES**							
^*^Reznik et al. (2020) USA	Healthy human with mild- moderate depression (*n* = 24)	Right frontotemporal area	Between-subjects with placebo control groups (no power administered)	Frequency: 0.5 MHz Intensity: 71 mW/cm^2^(*I*_SPTA_) Intensity: 14 W/cm^2^(*I*_SPPA_) PRF: 40 Hz DC: 0.26% PD: 65 μs SD: 30-s	Self-report assessments for mood and depression	Inhibitory (indirectly measured)	5 days of TUS sessions at the right frontotemporal area significantly improved worry and positive mood scores compared to the control group.
^*^Sanguinetti et al. (2020) USA	Healthy human (*n* = 48)	Right inferior frontal gyrus (rIFG)	Between-subjects with sham control groups (double-blind study)	Frequency: 0.5 MHz Intensity: 130 mW/cm^2^(*I*_SPTA_) Intensity: 54 W/cm^2^(*I*_SPPA_) PRF: 40 Hz DC: 0.26% PD: 65 μs Pulse repetition period:25 ms SD: 30-s	Mood states by Visual Analog Mood Scales (VAMS)	Inhibitory (indirectly measured)	TUS of rIFG significantly induced positive mood effects for up to 30 min compared to placebo control condition.
^*^Sanguinetti et al. (2020) USA	Healthy human (*n* = 9)	Right inferior frontal gyrus (rIFG)	Uncontrolled trial	Frequency: 0.5 MHz Intensity: 0.27 W/cm^2^(*I*_SPTA_) Intensity: 54 W/cm^2^(*I*_SPPA_) PRF: 40 Hz DC: 0.5% PD:65 μs Pulse repetition period:25 ms SD: 2 min	Mood states by VAMS; Resting-state fMRI (T3)	Inhibitory	(1) TUS of rIFG significantly induced positive mood effects 30 min after stimulation compared to baseline score. (2) TUS modulated on brain networks related to the area of sonication lasted up to 20 min. Twenty min of TUS at rIFG significantly reduced connectivity between rIFG and the related network as well as DMN connectivity.

*TUS, transcranial ultrasound stimulation; EMG, electromyography; I_SPTA_, spatial peak-temporal average intensity; I_SPPA_, spatial peak-pulse average intensity; PRF, pulse repetition frequency; DC, duty cycle; SD, sonication duration; PD, pulse duration; TBD, tone burst duration; ISI, inter-stimulus interval; Psptp, spatial-peak temporal-peak acoustic Pressure; LFP, Local filed potential; CBF, cortical blood flow; TMAS, Transcranial magneto-acoustic stimulation; PTZ, pentylenetetrazol; MB, microbubble; MPa, acoustic pressure*.

* *indicates human studies*.

Different intensity levels were used including 0.20, 0.40, 0.48, 0.53, 0.80, and from 1.10 to 2.11 W/cm^2^_(_*I*_SPPA)_ and frequencies in the range of 0.18–0.62 MHz. LI-TUS is capable of eliciting a motor response (Cui et al., 2019, 2020; Kim et al., 2019b; Wang et al., 2019a,c,d; Yuan et al., 2020) and inducing cortico-muscular synchronization at specific frequency bands of 5–150 Hz (Wang et al., 2019d). For example, Wang et al. (2019d) showed that alpha and beta synchronization appeared at 0.20 and 0.40 W/cm^2^ (*I*_SPPA_) while gamma synchronization occurred at 0.80 and 1.1 W/cm^2^_(_*I*_SPPA)_ stimulation. Furthermore, Yuan et al. (2020) reported a monotonic dose-response parametric relationship with the cerebral blood flow peak value increasing as a function of stimulation intensity [0.20, 0.40, 0.80, and 1.10 W/cm^2^ (*I*_SPPA_)] and SD (50, 100, 200, 300, and 400 ms), while DC (10, 20, 30, and 40%) only had a weak effect on peak response. These findings demonstrated the permutations of LI-TUS parameters that can manipulate modulatory function that is reflected as changes in the excitability of neuronal activity in M1 of healthy mice (Wang et al., 2019b).

Two studies examined the neuromodulation effect on the S1 with wide-field optical imaging (Choi et al., 2019) and near infrared spectroscopy (NIRS) measurements (Kim et al., 2019a). Both of the studies showed that neuronal calcium signaling was at least twice that observed in peripheral regions. Active ultrasonic stimulation at S1 with 1.077 and 0.468 W/cm^2^ (*I*_SPTA_), induced elevation of oxyhemoglobin (HbO) and a reduction in deoxyhemoglobin (RHb). Higher TUS intensity results in greater hemodynamic changes in S1 in healthy mice. Choi et al. (2019) showed that periodic pulse repetition in S1 with 0.662 W/cm^2^ (*I*_SPTA_) suppresses cortical activities in the auditory cortex (A1). That finding is significant given literature claims that the LI-TUS effects may be confounded by indirect stimulation of the auditory pathways in animals (Guo et al., 2018; Sato et al., 2018). For example, in a mouse model, Sato *et al* applied LI-TUS over A1 and S1 and visual cortex (V1) as a control non-auditory brain region to examine the direct/indirect effect of LI-TUS changes in activation of auditory pathways. They showed that bilateral activation of the A1 was elicited by the pure tone of ultrasound. Interestingly, V1 activation also remarkably matched the auditory activity when LI-TUS was applied over V1. Thus, this study showed strong activation of A1 elicited by indirect application of LI-TUS at different brain regions, which is in line with previous studies (Guo et al., 2018; Sato et al., 2018).

Several studies targeted neural regions beyond M1 and S1. For example, LI-TUS stimulation of V1 resulted in similar outcomes occurring in the S1 (Choi et al., 2019). Specifically, LI-TUS generated a 100% increase in neuronal calcium signaling compared to that seen in the peripheral regions. Also, both periodic and random pulse repetition rate stimulation used in V1 suppressed cortical activities in A1 (Choi et al., 2019). Wang et al. (2020a) demonstrated that use of LI-TUS on the midbrain periaqueductal gray (PAG) can modulate defensive behaviors in healthy mice, that is, stimulation of PAG resulted in increased engagement of location-specific passive avoidance behavior and faster movement time compared to sham stimulation group. The modulatory effect of LI-TUS in the thalamus was investigated using rodent and sheep models. For example, Darrow et al. (2019) found that the somatosensory evoked potential (SSEP) waveform was suppressed when LI-TUS was focused on the ventral posterolateral nucleus (VPL) contralateral to the stimulated tibial nerve in rodents (Darrow et al., 2019). Pang et al. (2020) stimulated the hypothalamus of aging mice using two different PRF (1,000 Hz or 10 Hz). Stimulation with the lower frequency at 10 Hz led to significantly superior movement and learning compared to those treated with 1,000 Hz or sham stimulation (Pang et al., 2020). Yoon et al. (2019) modulated the left thalamus and M1 in sheep with four different DC percentages and four different tone burst durations (TBD). They found that a DC of 70% and 0.5 ms TBD outperformed other parameters but the overall effect was transient and reversible (Yoon et al., 2019).

Four studies investigated the neuromodulatory effects of LI-TUS in the amygdala, anterior cingulate cortex (ACC) (Folloni et al., 2019; Fouragnan et al., 2019; Khalighinejad et al., 2020), supplementary motor area (SMA) and frontopolar cortex (FPC) (Verhagen et al., 2019) in healthy macaque's brain using functional magnetic resonance imaging (fMRI). All four studies followed a similar procedure in which 40 s of stimulation was delivered using 250 kHz frequency that was pulsed at 10 Hz with repetitive 30 ms bursts of ultrasound every 100 ms. This protocol ensured that the effect of LI-TUS impacted the macaque's brain regions of interest without auditory confounds up to 2 h following stimulation (Verhagen et al., 2019). Folloni et al. (2019) found that LI-TUS stimulation in the amygdala and ACC resulted in focal modulation of neuronal activation in these regions. Based on resting-state fMRI connectivity analysis, the macaques in the control that did not receive LI-TUS demonstrated strong interconnectivity between the amygdala and ACC with the rest of the brain. However, after repetitive LI-TUS, the activity in the targeted structures and the connectivity with their respective networks throughout the brain were decreased, with the effects lasting >1 h and unmediated by auditory confounds (Folloni et al., 2019). Khalighinejad et al. (2020) stimulated the basal forebrain (BF) using TUS and found that there was enhanced activity between the BF and ACC but suppressed with every other region (Khalighinejad et al., 2020). Fouragnan et al. (2019) showed that ACC stimulation with LI-TUS induced negative behavioral changes in macaques by reducing ability and accuracy in performance and the effect was also reversible (Fouragnan et al., 2019). The impact of LI-TUS stimulation on the neuronal connectivity pattern of SMA and FPC was also investigated. Verhagen et al. (2019) showed that LI-TUS enhanced the connectivity patterns between SMA and proximal areas but reduced the coupling between SMA and distal regions. Moreover, FPC connectivity with the dorsomedial and lateral prefrontal cortex (PFC) was enhanced whereas connectivity with the lateral orbitofrontal cortex and other regions of PFC was reduced (Verhagen et al., 2019). Lastly, the impact of LI-TUS stimulation on forming decision behavior of frontal eye fields (FEF) was investigated in healthy macaques. In this study, LI-TUS delivered ultrasound waves at 270 kHz pulsed at 500 Hz PRF in 300 ms stimulus duration. Behavioral data revealed that targeting the left FEF increased the proportion of rightward choices, whereas, stimulation of the right FEF increased the proportion of leftward choices. As an active control, region-specific LI-TUS applied at M1 failed to elicit significant biases in choice behavior (Kubanek et al., 2020).

The neuromodulatory effect of LI-TUS has been investigated in disease animal models. Relatively higher intensity of 5.10 W/cm^2^_(_*I*_SPPA)_, compared to that used in healthy mice, was used in a Parkinson's disease mouse model to elicit M1 excitability. These data revealed similar LFP signal compared to that observed in the healthy control group (Wang et al., 2020a). Behavioral outcome associated with immunofluorescence technique measurements indicated that after 4 days of LI-TUS at M1 with 0.76 W/cm^2^_(_*I*_SPPA)_ in PD mice showed an increase of c-Fos positive cells in M1 as well as superior behavioral performance compared to the sham control group (Zhou et al., 2019a). When the substantia nigra (SN) was stimulated (Zhou et al., 2019b), the stimulated healthy mice did not exhibit significantly different behavior compared to the control mice. However, stimulation of SN in PD mice did induce significant improvement in spatial related forms of motor behavior performance than the control group. Furthermore, locomotor functions in PD mice were significantly improved after 10-day administration of non-focused TUS treatment at 0.30 W/cm^2^ over 5 min. Specifically, administration of LI-TUS treatment with these parameters induced more dopamine (DA) release in the striatum compared to that in untreated PD mice (Xu et al., 2020). LI-TUS has also been shown to be applicable in the setting of epilepsy management. Chen et al. (2020) showed that acute epileptic neuronal activity in rats was significantly suppressed by LI-TUS that targeted the cortex, hippocampus, and thalamus regions (MI 0.75, DC 30%, and 600 s of total exposure time) (Chen et al., 2020). In a study with monkeys, Zou et al. (2020) applied LI-TUS at a frequency of 800 kHz directed at the PFC resulting in significantly reduced number of seizures and increased inter-seizure interval time than in the sham group (Zou et al., 2020).

### Neuromodulatory Effect of LI-TUS in Humans

Only two human studies met the inclusion criteria. The studies used the same frequency and pulse duration but the DC and intensity were different (Table 1). Sanguinetti et al. (2020) showed that modulating right inferior frontal gyrus (rIFG) activity with LI-TUS can induce positive mood effects. A resting-state fMRI was used to follow the functional connectivity changes between rIFC and other neural regions (e.g., subgenual cortex, orbitofrontal cortex, inferior prefrontal gyrus, dorsal anterior cingulate cortex, and entorhinal cortex) that are involved in emotional regulation (Sanguinetti et al., 2020). Similarly, Reznik et al. (2020) found that stimulation of the right frontotemporal area in subjects with depression significantly improved their worry and positive mood scores compared to the control group (Reznik et al., 2020). The indirect effect of auditory confound might be influenced by the precise investigation of the effect of LI-TUS. This finding is in line with the recent study where Braun et al. (2020) found that LI-TUS in humans has auditory confounds (Braun et al., 2020).

### Methodological Quality

For the 24 animal studies, the SYRCLE scores were yes (32.1%), no (42.9%), and unclear (25%) (Supplementary Table 2). The two human studies evaluated by PEDro had a low risk of bias (Supplementary Table 3). For the Sanguinetti et al. (2020) study, the NIH tool revealed a moderate to high level of reliability (Supplementary Table 4).

## Discussion

Low-intensity ultrasound stimulation (LI-TUS) has received considerable attention for scientific investigation in neuromodulation, especially in the animal domain. In this systematic review, we summarize the findings reported from 26 studies regarding the effect of LI-TUS in neuromodulation in both animals and human studies published from January 2019 to June 2020. While LI-TUS showed excitatory effects in animal studies, this excitatory effect has not been observed in primate and human studies. Different sonication parameters likely resulted in divergent neuromodulation responses. Regarding the ultrasound beam profile, it is arguable that the most critical parameter difference between focused LI-TUS and unfocused LI**-**TUS is the volume of the brain tissue impacted, which can lead to differential stimulation effect (Di Biase et al., 2019). For example, administration of focused LI-TUS at M1 with frequency at 0.50 MHz and intensity at 17.12 W/cm^2^ (*I*_SPPA_) and 6.16 W/cm^2^ (*I*_SPTA_) inhibits neuronal excitability in humans (Legon et al., 2018). However, unfocused TUS at M1 required a higher frequency of 2.32 MHz and intensity of 34.96 W/cm^2^ (*I*_SPPA_) and 132.85 mW/cm^2^ (*I*_SPTA_) to induce neuronal excitability measured by motor evoked potential amplitude (Gibson et al., 2018). Lemaire et al. (2019) reported that the excitation thresholds of neurons are sensitive to parameters based on a multi-scale optimized neuronal intramembrane cavitation model from which they found that frequency above 1 MHz likely reduces neuronal excitability (Lemaire et al., 2019). This was not true for both animal and human studies included in this review as seven out of eight animal studies used frequency below 1 MHz and observed inhibitory effects. The two human studies used 0.50 MHz and observed inhibitory effects. This suggests that parameters other than frequency also play a role in neuromodulatory effects (Table 2), and the outcome might be dependent on disease conditions and location.

**Table 2:** Summary of key ultrasonic parameters by neuromodulatory effect.

**Study**		**Frequency (MHz)**		**Intensity (W/cm**^****2****^**)**		**DC (%)**	**SD (ms)**
		** <1 MHz**	**≥1 MHz**	***I*_**SPTA**_**	***I*_**SPPA**_**		
Inhibitory	Animal	0.25 (*n* = 5), 0.5, 0.75, 0.8	3.2	0.7, 1.43, 2.81, 5.6, 5.63, 6.4, 7.2, 9.5, 19.5, 20	15.8, 18.2, 18.9, 21.2, 119.8	5, 8, 20, 30 (*n* = 5), 36, 50, 70 (*n* = 2)	100, 200, 40 s (*n* = 4)
Excitatory	Animal	0.18, 0.25, 0.5 (*n* = 5), 0.62 (*n* = 2), 0.8	1 (*n* = 3), 3.8, 8, 10	0.06 (*n* = 2), 0.07, 0.29, 0.47, 0.54, 0.66, 1.8	0.1, 0.14, 0.2 (*n* = 4), 0.3, 0.4, 0.48 (*n* = 2), 0.53, 0.76, 0.8, 1.1 (*n* = 3), 2.11 (*n* = 2), 5.4, 15.8, 18.2	9, 10 (*n* = 4), 20 (*n* = 4), 30 (*n* = 2), 40 (*n* = 3), 50 (*n* = 7), 70, 90	50 (*n* = 2), 100, 120, 200 (*n* = 6), 300 (*n* = 2), 400 (*n* = 7), 1 s,6 s
Inhibitory	Human	0.5 (*n* = 2)		0.071, 0.13, 0.27	14, 54 (*n* = 2)	0.26 (*n* = 2), 0.5	30 s (*n* = 2), 2 min

*I_SPTA_, spatial peak-temporal average intensity; I_SPPA_, spatial peak-pulse average intensity; DC, duty cycle; SD, sonication duration; PD, pulse duration*.

Several primate studies using repetitive LI-TUS hold promising potential for therapeutic application. For example, LI- TUS can modulate neuronal plasticity and can induce alterations in functional connectivity patterns (Folloni et al., 2019; Fouragnan et al., 2019; Verhagen et al., 2019; Khalighinejad et al., 2020). Khalighinejad et al. (2020) showed that stimulation of BF resulted in significant integration between BF and ACC, while widespread suppression of connectivity between BF and other areas occurred. This effect was not observed in other stimulated brain regions (e.g., SMA) in primates (Verhagen et al., 2019). This was found because disruption of the neuronal circuitry of BF affects the entire hemisphere due to its extensive connectivity with different regions of the brain. As a consequence of the delivery of repetitive LI-TUS to BF and ACC, behavioral changes resulted in disruption of decision-making behavior, possibly due to the influence of repetitive LI-TUS on modulation of targeted brain activity (Fouragnan et al., 2019; Khalighinejad et al., 2020). In human, LI-TUS has been used for treatment of mood disorders such as depression (Reznik et al., 2020; Sanguinetti et al., 2020). In summary, it is encouraging that these exciting observations have been noticed but the robustness of these effects from LI-TUS needs to be further demonstrated. Despite numerous investigations of LI-TUS as a viable neuromodulatory tool, many scientific questions remain. For example, what is the underlying mechanism of the mechanical bioeffects from LI-TUS? What are the optimal parameters to be used in neurologically impaired subjects? The optimal frequency of LI-TUS for effective neuromodulation remains largely unknown. Due to the differences in skull size and geometry of the brain, the parameters from LI-TUS used in animals are less likely to be translated to humans. Computational models may aid the translational process.

Four recent studies reported that indirect stimulation of auditory pathways may interfere with the neuromodulatory effects of LI-TUS (Guo et al., 2018; Sato et al., 2018; Mohammadjavadi et al., 2019; Braun et al., 2020). Although the impact of auditory confounding can be minimized *via* delivery of an external auditory waveform, it is important to recognize its existence as it may influence neuromodulatory outcomes. Further research efforts are needed in order to elucidate the mechanism of auditory confound to better control for such effect in future studies.

LI-TUS is non-invasive and likely safe to human application. For example, a group of investigators at the University of Minnesota enrolled 120 participants in seven LI-TUS human studies, of which 64 subjects (53%) responded to a follow-up questionnaire surveying adverse events from stimulation. None of the participants reported serious adverse events (Legon et al., 2020). Although, a dedicated investigation of safety profiles of LI-TUS in humans are still lacking (Pasquinelli et al., 2019). Safety profiles must be treated with high priority in human studies and need to be systematically investigated, especially in subjects with neurological impairments.

## Conclusion

LI-TUS appears to be a promising tool for neuromodulation that can target both superficial and deep structures of the brain to induce cognitive and/or motor behavioral changes. There are an increasing number of publications using this brain modulatory tool for various neuropsychiatric disorders, however, a majority of studies are still limited to the animal domain with only two studies in the human domain in the last 18 months. Safety of TI-TUS appears reasonable, but dedicated and systematic investigations of safety studies are still required. Future studies focused on optimizing parameters of LI-TUS (i.e., intensity, frequency, SD, and DC) for disease-specific conditions are needed in order to successfully translate into clinical use in the near future.

## Author Contributions

TK and WF formulated the project. TK and CP performed the review and drafted the manuscript. PC, JF, BM, CN, PW, MC, and XJ contributed to critical writing and revision of the manuscript. All authors contributed to the article and approved the submitted version.

## Conflict of Interest

The authors declare that the research was conducted in the absence of any commercial or financial relationships that could be construed as a potential conflict of interest.
