# Supplementary material for: Effect of Low Intensity Transcranial Ultrasound Stimulation on Neuromodulation in Animals and Humans: An Updated Systematic Review
Source: Front Neurosci. 2021 Apr 14;15:620863. doi: 10.3389/fnins.2021.620863 (PMC8079725; doi:10.3389/fnins.2021.620863)
Supplement: Supplementary file 1 [file Table_1.pdf]

Supplementary materials

Effect of Low Intensity Transcranial Ultrasound Stimulation on Neuromodulation in Animals and Humans: An Updated Systematic Review

Taewon Kim[1], Christine Park[1], Pratik Y. Chhatbar[1], Jody Feld[2], Brian Mac Grory [1], Chang S. Nam[3], Pu Wang[4], Mengyue Chen[5], Xiaoning Jiang[5] and Wuwei Feng[1]*

[1] Department of Neurology, Duke University School of Medicine, Durham, NC, United States, [2] Physical Therapy Division, Department of Orthopaedic Surgery, Duke University School of Medicine, Durham, NC, United States, [3] Fitts Department of Industrial and Systems Engineering, North Carolina State University, Raleigh, NC, United States, [4] Department of Rehabilitation Medicine, Seventh Affiliated Hospital, Sun Yat-Sen University, Shengzhen, China, [5] Department of Mechanical and Aerospace Engineering, North Carolina State University, Raleigh, NC, United States

Table S1. Database search strategy

Table S2. Quality Assessment of Included Animals Studies by SYRCLE's tool

Table S3. Quality Assessment of Included Human Studies by PEDro

Table S4. Quality Assessment of Included Human Studies by NIH tool

Supplementary table 1. Database search strategy

Database	Search Terms
**Pubmed**	(((transcranial[All Fields] AND ("diagnostic imaging"[Subheading] OR ("diagnostic"[All Fields] AND "imaging"[All Fields]) OR "diagnostic imaging"[All Fields] OR "ultrasound"[All Fields] OR "ultrasonography"[MeSH Terms] OR "ultrasonography"[All Fields] OR "ultrasound"[All Fields] OR "ultrasonics"[MeSH Terms] OR "ultrasonics"[All Fields])) OR (transcranial[All Fields] AND focused[All Fields] AND ("diagnostic imaging"[Subheading] OR ("diagnostic"[All Fields] AND "imaging"[All Fields]) OR "diagnostic imaging"[All Fields] OR "ultrasound"[All Fields] OR "ultrasonography"[MeSH Terms] OR "ultrasonography"[All Fields] OR "ultrasound"[All Fields] OR "ultrasonics"[MeSH Terms] OR "ultrasonics"[All Fields]))) OR (("diagnostic imaging"[Subheading] OR ("diagnostic"[All Fields] AND "imaging"[All Fields]) OR "diagnostic imaging"[All Fields] OR "ultrasound"[All Fields] OR "ultrasonography"[MeSH Terms] OR "ultrasonography"[All Fields] OR "ultrasound"[All Fields] OR "ultrasonics"[MeSH Terms] OR "ultrasonics"[All Fields]) AND stimulation[All Fields])) AND ("Neuromodulation"[Journal] OR "neuromodulation"[All Fields]) AND ("2019/01/01"[PDAT] : "2020/12/31"[PDAT])
**Embase**	('transcranial ultrasound'/exp OR 'transcranial ultrasound' OR (transcranial AND ('ultrasound'/exp OR ultrasound)) OR 'transcranial focused ultrasound'/exp OR 'transcranial focused ultrasound' OR (transcranial AND focused AND ('ultrasound'/exp OR ultrasound)) OR 'ultrasound stimulation' OR (('ultrasound'/exp OR ultrasound) AND ('stimulation'/exp OR stimulation))) AND (Neuromodulation /exp OR Neuromodulation) AND [2019-2020]/py
**MEDLINE**	((transcranial ultrasound or transcranial focused ultrasound or ultrasound stimulation) and Neuromodulation).mp. [mp=title, abstract, original title, name of substance word, subject heading word, floating sub-heading word, keyword heading word, organism supplementary concept word, protocol supplementary concept word, rare disease supplementary concept word, unique identifier, synonyms] (yr="2019 - 2020")
**Web of Sci**	(transcranial ultrasound OR transcranial focused ultrasound OR ultrasound stimulation) AND TOPIC: (neuromodulation)<br>Timespan: 2019-2020

Assessing the methodological quality of included studies

The SYRCLE's Risk of Bias tool aims to address the following biases: selection bias, performance bias, detection bias, attrition bias, and reporting bias. The answer spectrum indicates "Yes" for low risk of bias, "No" for high risk of bias, and "Unclear" for unclear risk of bias if insufficient details were reported (see table S2). The PEDro scale is composed of the following 10 items: 1) random allocation; 2) concealed allocation; 3) similarity at baseline; 4) subject blinding; 5) therapist blinding; 6) assessor blinding; 7) > 85% follow up for at least one key outcome; 8) intention-to-treat analysis; 9) between-group statistical comparison for at least one key outcome; and 10) point and variability measures for at least one key outcome. The answer spectrum on the PEDro indicates either a score of (1) present or (0) absent with a total score of 10. A score of ≥ 6/10 is considered moderate to high quality (see table S3). Uncontrolled or single-case trial studies were assessed by the National Institutes of Health (NIH) quality assessment tool. [1] Three rating qualities (Good, Fair and Poor) determine the degree of risk of bias (see table S4).

Supplementary table 2. Quality Assessment of Included Animals Studies by SYRCLE's tool

STUDY	SELECTION BIAS			PERFORMANCE BIAS		DETECTION BIAS		ATTRITION BIAS	REPORTING BIAS	OTHER
	**Sequence generation**	**Baseline characteristics**	**Allocation concealment**	**Random housing**	**Blinding**	**Random outcome assessment**	**Blinding**	**Incomplete outcome data**	**Selective outcome reporting**	**Other sources of bias**
Chen et al 2020	yes	yes	no	no	no	no	yes	unclear	unclear	yes
Choi et al 2019	yes	yes	no	unclear	unclear	unclear	unclear	yes	yes	yes
Cui et al 2019	no	unclear	no	unclear	no	no	no	yes	unclear	yes
Cui et al 2020	no	unclear	no	no	no	no	no	yes	no	yes
Darrow et al 2019	no	no	no	no	no	no	unclear	unclear	unclear	yes
Folloni et al 2019	yes	yes	no	no	no	no	no	yes	unclear	yes
Fouragnan et al 2019	yes	yes	no	no	no	no	no	yes	unclear	yes
Khalighinejad et al 2019	yes	yes	no	no	no	no	no	yes	unclear	yes
Kim. E et al 2019	unclear	yes	no	unclear	unclear	no	no	yes	unclear	yes
Kim. H et al 2019	unclear	unclear	no	unclear	unclear	no	no	yes	yes	yes
Kubanek et al 2020	unclear	yes	no	no	no	no	no	yes	unclear	yes
Pang et al 2020	unclear	no	no	no	no	no	no	unclear	no	yes
Verhagen et al 2019	yes	yes	no	no	no	no	no	yes	unclear	yes
Wang. H et al 2019	yes	yes	no	unclear	no	no	no	yes	unclear	yes
Wang. X et al 2019	unclear	yes	no	unclear	no	no	no	yes	unclear	yes
Wang. Y et al 2019	unclear	yes	no	unclear	no	no	no	yes	unclear	yes
Wang. Z et al 2019	unclear	yes	no	unclear	no	unclear	no	yes	unclear	yes
Wang. Y et al 2020	unclear	no	no	unclear	no	no	no	yes	unclear	yes
Xu et al 2020	unclear	no	no	unclear	no	no	no	yes	unclear	yes
Yoon et al 2019	unclear	yes	no	unclear	no	no	no	yes	yes	yes
Yuan et al 2020	unclear	yes	no	unclear	no	no	no	yes	yes	yes
Zou et al 2020	yes	yes	no	no	no	no	yes	no	unclear	yes
Zhon. X et al 2019	unclear	yes	no	unclear	no	unclear	no	yes	yes	yes
Zhon. H et al 2019	unclear	yes	no	unclear	no	no	unclear	yes	yes	yes

Supplementary table 3. Quality Assessment of Included Human Studies by PEDro

Study	Random allocation	Concealed allocation	Baseline comparability	Blind subjects	Blind therapists	Blind assessors	Adequate follow-up	Intention -to-treat analysis	Between group comparisons	Point estimates and variability	Total Scores
Sanguinetti et al 2020 (a)	1	0	1	1	1	1	1	1	1	1	9/10
Reznik et al 2020	1	0	1	1	1	1	1	1	1	1	9/10

Supplementary table 4. Quality Assessment of Included Human Studies by NIH tool

**Q. The National Institutes of Health (NIH) quality assessment tool for before-after (Pre-Post) study with no control group**			
Sanguinetti et al 2020 (b)	Response options: Yes, No, cannot determine (CD), not applicable (NA), not reported (NR)		
1. Was the study question or objective clearly stated?	**Yes**		
2. Were eligibility/selection criteria for the study population prespecified and clearly described?		**No**	
3. Were the participants in the study representative of those who would be eligible for the test/service/intervention in the general or clinical population of interest?		**No**	
4. Were all eligible participants that met the prespecified entry criteria enrolled?	**Yes**		
5. Was the sample size sufficiently large to provide confidence in the findings?		**No**	
6. Was the test/service/intervention clearly described and delivered consistently across the study population?	**Yes**		
7. Were the outcome measures prespecified, clearly defined, valid, reliable, and assessed consistently across all study participants?	**Yes**		
8. Were the people assessing the outcomes blinded to the participants' exposures/interventions?	**Yes**		
9. Was the loss to follow-up after baseline 20% or less? Were those lost to follow-up accounted for in the analysis?	**Yes**		
10. Did the statistical methods examine changes in outcome measures from before to after the intervention? Were statistical tests done that provided p values for the pre-to-post changes?	**Yes**		
11. Were outcome measures of interest taken multiple times before the intervention and multiple times after the intervention (i.e., did they use an interrupted time-series design)?			**NR**
12. If the intervention was conducted at a group level (e.g., a whole hospital, a community, etc.) did the statistical analysis take into account the use of individual-level data to determine effects at the group level?	**Yes**		
**Quality Rating**	**Good**	Fair	Poor

frontiers
